# Acute kidney injury and cardiac surgery: impact of fluid balance on AKI classification and prognosis

**DOI:** 10.1186/cc13557

**Published:** 2014-03-17

**Authors:** EM Moore, A Tobin, D Reid, J Santamaria, R Bellomo

**Affiliations:** 1 Monash University, Melbourne, Australia; 2 St Vincent's Hospital, Melbourne, Australia

## Introduction

We assessed the effect of fluid balance (FB) on acute kidney injury (AKI) classification/prognosis in cardiac surgical patients by comparing patients classified with AKI, before and after adjusting the creatinine (used to classify AKI) for FB. Fluid accumulation is associated with negative outcomes including development of AKI in critically ill patients [1]. Cardiac surgical patients commonly receive large volumes of fluid postoperatively and could be at risk for the harmful effects of fluid accumulation. Furthermore, fluid accumulation may influence serum creatinine concentration and mask AKI [2].

## Methods

We performed a retrospective analysis of prospectively collected data on all cardiac surgical patients admitted to St Vincent's Hospital ICU, Melbourne, Australia from 1 July 2004 to 30 June 2012. AKI Network creatinine criteria were used to classify AKI in the usual method and then using FB-adjusted creatinine (FB at 18 hours and an assumption that total body water is 60% of weight involved). FB (total i.v. input minus (total urine output + chest drain losses)) was calculated for 18 hours post surgery as most patients were in the ICU for this period.

## Results

Patients classified with AKI increased from 27.7% to 37.2% (*n *= 2,171) after adjusting creatinine for FB. Patients were categorised into four groups based on presence or absence of AKI before and after adjustment for FB: group A, no AKI before or after adjustment for FB; group B, no AKI before/AKI after; group C, AKI before/no AKI after; and group D, AKI before and after. Group B (*n *= 209) had an in-hospital mortality rate similar to patients in group D (*n *= 599) (3.4% vs. 4.3%, *P *= 0.53) and greater than those in group A (*n *= 1,333) (3.4% vs. 1.6%, *P *= 0.07). Group B also had an ICU mortality rate similar to patients in group D (2.9% vs. 2.7%, *P *= 0.88) and significantly greater than those in group A (2.9% vs. 0.7%, *P *= 0.003). The need for renal replacement therapy (RRT) in group B was also high as for patients in group D (7.7% vs. 12.4%, *P *= 0.06) and was significantly greater than those in group A (7.7% vs. 1.6%, *P *< 0.001). Thus, hospital and ICU mortality and use of RRT in patients classified with AKI only after adjustment for FB were similar to patients with AKI before and after adjustment for FB and were notably higher than those of patients without AKI.

## Conclusion

Lack of adjustment for FB post cardiac surgery may mask the presence of AKI that is associated with increased risk for death and RRT, which could hinder optimal treatment.
